# Irisin levels in *LMNA*-mutated lipodystrophic syndromes

**DOI:** 10.1186/1750-1172-10-S2-O21

**Published:** 2015-11-11

**Authors:** Faiza Bensmaine, Marie-Christine Vantyghem

**Affiliations:** 1 Endocrinology and Metabolism Department, Lille Nord de France University Hospital, Lille, France; 2 Inserm U1190, Lille University Hospital, Lille, France

## 

Sarcopenia is defined by decreased muscle mass and impaired muscle function, which may be associated with frailty and eventually higher mortality rates. It is physiologically induced by aging but also related to obesity by different mechanisms such as 1) diminished physical activity; 2) elevated oxidative stress; 3) inflammatory cytokines; 4) increased catabolic state, through hypothalamo-pituitary axis; 5) muscle insulin resistance; 6) abnormal muscle progenitor cells differentiation to an adipocyte-like phenotype as a result of paracrine signals from (adipo)cytokines.

Adipose tissue is classified as white adipose tissue (WAT), the major energy storing tissue, brown adipose tissue (BAT), which mediates non-shivering thermogenesis and brite adipocytes (brown in white). Increasing BAT and energy expenditure in adult humans could be a therapeutic strategy to combat obesity. Brown adipocytes are thought to originate from a precursor shared with skeletal muscle that expresses Myf5-Cre, while white adipocytes originate from a Myf5-negative precursors. This provides a rational explanation to why BAT is more metabolically favorable than WAT, even if the situation is more complex because subsets of white adipocytes also arise from Myf5-Cre expressing precursors. Differences in origin between adipocytes could explain metabolic heterogeneity between depots and/or influence body fat patterning particularly in lipodystrophic disorders.

Irisin is a newly discovered myokine, associated with ‘browning’ of the WAT. It displays a day-night rhythm, is correlated with lean body mass, and increases after exercise in healthy young individuals, despite an association with major adverse cardiovascular events and polycystic ovary disease[1]. Deficiency of myostatin, and thus stimulation of muscle growth, has also been reported to induce irisin and its precursor FNDC5 expression in muscle and drive the browning of WAT in mice.

Familial partial lipodystrophy, Dunnigan variety (FPLD2), an autosomal dominant disorder caused by *LMNA* mutations, is characterized by fat loss from the extremities and apparent muscular hypertrophy. However, it is unclear whether these patients appear muscular because of a lack of subcutaneous fat or have an actual increase in muscle mass[2]. Moreover adipose tissue mitochondrial dysfunction triggers a lipodystrophic syndrome with insulin resistance, hepatosteatosis, and cardiovascular complications[3,4].

Therefore, our objective was to identify the status of lean mass in *LMNA*-mutated lipodystrophic syndromes and to determine simple biomarkers to differentiate *LMNA*-mutated and acquired lipodystrophies. To do so, we assessed the lean (as a surrogate of muscle mass) and fat mass with absorptiometry in FPLD2 patients, non-diabetic obese and control subjects using dual-emission x-ray absorptiometry and magnetic resonance imaging, and measured the myokine irisin and the adipokine leptin blood levels . Our hypothesis is that the rupture of balance between physiological lean and fat mass in lipodystrophic syndromes could explain the evolution towards insulin-resistance (Trial registration: Clin.gov2009-AO-1169-48/PHRC2009 09/094).
